# Emerging Role of Metabolomics in Ovarian Cancer Diagnosis

**DOI:** 10.3390/metabo10100419

**Published:** 2020-10-19

**Authors:** Asia Saorin, Emanuela Di Gregorio, Gianmaria Miolo, Agostino Steffan, Giuseppe Corona

**Affiliations:** 1 Immunopathology and Cancer Biomarkers Unit, Centro di Riferimento Oncologico di Aviano (CRO), IRCCS, 33081 Aviano, Italy; asaorin@cro.it (A.S.); emanuela.digregorio@cro.it (E.D.G.); asteffan@cro.it (A.S.); 2 Medical Oncology and Cancer Prevention Unit, Centro di Riferimento Oncologico di Aviano (CRO), IRCCS, 33081 Aviano, Italy; gmiolo@cro.it

**Keywords:** metabolomics, ovarian cancer, biomarkers, diagnosis

## Abstract

Ovarian cancer is considered a silent killer due to the lack of clear symptoms and efficient diagnostic tools that often lead to late diagnoses. Over recent years, the impelling need for proficient biomarkers has led researchers to consider metabolomics, an emerging omics science that deals with analyses of the entire set of small-molecules (≤1.5 kDa) present in biological systems. Metabolomics profiles, as a mirror of tumor–host interactions, have been found to be useful for the analysis and identification of specific cancer phenotypes. Cancer may cause significant metabolic alterations to sustain its growth, and metabolomics may highlight this, making it possible to detect cancer in an early phase of development. In the last decade, metabolomics has been widely applied to identify different metabolic signatures to improve ovarian cancer diagnosis. The aim of this review is to update the current status of the metabolomics research for the discovery of new diagnostic metabolomic biomarkers for ovarian cancer. The most promising metabolic alterations are discussed in view of their potential biological implications, underlying the issues that limit their effective clinical translation into ovarian cancer diagnostic tools.

## 1. Introduction

Ovarian cancer (OC) is the third most common gynecologic malignancy worldwide and the fifth cause of cancer death among women [1], with a number of diagnosed new cases equal to 300,000 in 2018 [2]. The term OC includes more than 30 different histotypes [3]; among them, epithelial ovarian cancer (EOC) is the most common, accounting for about 90% of the total [4].

About 63% of OC patients are diagnosed at Stage IV cancer, and only 27.6% survive at 5 years. The poor prognosis is mainly due to late diagnoses, since early detection at Stage I significantly improves the survival rate, i.e., to 92.4% [1]. The delay in diagnosis is principally associated with the disease characteristics, because it remains quite asymptomatic until it metastasizes [5]. 

Transvaginal ultrasonography and blood CA-125 levels are the current diagnostic strategies for OC, but present important limitations. Transvaginal ultrasonography can mistake cancer for functional cysts, especially in premenopausal women, while CA-125 has a high false-positive rate, revealing only 50% of stage I cases [6,7,8]. The poor diagnostic power of the current tests highlights the need of novel diagnostic biomarkers, especially for early stage detection.

A great contribution in this regard has been made by the application of the *omics* sciences, represented by genomics, transcriptomics, proteomics and metabolomics. Their continuous advancement has contributed to the development of new medical approaches that better decipher the molecular changes underlying diseases and interpatient variability [9]. In this context, particularly noteworthy is the role of metabolomics [10], defined as the comprehensive study of all small-molecules (<1.5kDa) present in the biological system [11]. The metabolomics profile, unlike the genomics profile, reflects the biochemical events that occur in the organism as a result of the complex interactions among age, sex, gene transcription, protein expression, physio-pathological conditions including gut microbiome activity and environmental effects. Consequently, it offers a closer description of the patient’s disease phenotype, which is useful not only for diagnostic purposes, but also to understand the clinical outcome variability that is at the basis of precision medicine [12,13].

Metabolomics can reveal specific host metabolic alterations induced by the cancer, mainly to sustain its growth [14]. These alterations can be detected in biological fluids such as blood, plasma, urine and ascites, and knowledge of these systemic changes is considered a valid approach to discover new diagnostic and prognostic biomarkers [15]. Due to the urgency for novel OC biomarkers, the metabolomic approach has been widely applied in recent years. Despite the publication of numerous studies on the topic, real clinical benefit for OC diagnosis is yet to be achieved [16]. Differences among metabolomic studies and methodological issues have limited the potential use of the identified biomarkers for OC diagnosis [17]. 

In this critical review, we aim to update the status of metabolomics OC biomarkers research for diagnostic purposes. The most critical steps of metabolomics workflow are analyzed in an attempt to explain the potential pitfalls of metabolomics-based biomarkers research that often generate inconsistent results among studies and limit the effective translation of these findings to a clinical setting.

## 2. Metabolomics Workflow 

Metabolomic studies for the research of diagnostic biomarkers involve comparisons of the metabolomics profiles of two groups: a control group not affected by the investigated condition, and a test group that carries information about the analyzed condition [18]. Analyses of metabolomics profiles are generally performed by nuclear magnetic resonance (NMR) and mass spectrometry (MS) techniques, coupled with separation systems such as liquid (LC) or gas chromatography (GC). Targeted or untargeted approaches can be applied; the former refers to the detection of a known set of metabolites, while the latter consists of the detection of all metabolites present in a sample [19]. After metabolomic data acquisition, different statistical strategies of analysis make it possible to screen and select significantly altered metabolites linked to the biological context under study. 

NMR is a nondestructive, intrinsically quantitative method that does not require sample preparation. Compared with MS, it has higher reproducibility but lower sensitivity, i.e., mM concentrations. Conversely, MS is 10 fold more sensitive and selective, but batch and drift effects can generate biases in quantifications. In order to overcome these limits and obtain reliable quantitative data, labelled internal standards (IS) are added to samples during the pre-analytical phase [18]. Moreover, the inclusion of quality control samples (QC) along the analytical run makes it possible to evaluate the reproducibility and accuracy of the analytical process [19]. Therefore, the results of metabolomic investigations where IS and QC were included should be considered more reliable. 

The outcomes of metabolomics analyses consist of highly complex datasets that require pretreatment steps to reduce systematic biases. This process includes the normalization, scaling and transformation of the raw data [20]. Data normalization [21] and scaling [21,22] make samples and variables comparable to each other, while data transformation makes it possible to achieve a normal distribution which is suitable for parametric statistics [20]. The mathematical methods applied for these pretreatment steps depend on the data type, the biological question and the selected analytical methods [20]. For instance, in the case of urine samples, normalization to creatinine is commonly performed to eliminate the dilution effect due to different water intakes among patients [21,23], or in the case of an untargeted MS profile, data are normalized by total ion current (TIC) [24].

After data pretreatments, a statistical analysis is performed to reveal significant differences between case and control metabolomics profiles, highlighting metabolites which are potentially eligible for biomarker evaluations. A statistical analysis can be performed by both univariate and multivariate methods [21]. Multivariate unsupervised principal component analysis (PCA) and supervised partial least square discriminant analysis (PLS-DA) are commonly used to identify specific disease metabolomic signatures. PCA is particularly useful to have a first look of the dataset structure. The group space separation is obtained only when the intragroup variability is smaller than the intergroup variability. Since this latter feature is generally uncommon in samples derived from clinical studies, supervised methods such as PLS-DA can be applied to identify differences among groups. However, since the number of analyzed metabolites is often much larger than the sample size, supervised models can over-fit the data information [25] and need to be validated by internal cross validation and permutation approaches [26,27]. This over-fitting issue can be avoided by removing useless information, i.e., by the selection of the variables which are most correlated with the class membership [28] or by a better patient stratification strategy [23]. 

The metabolomic biomarkers specific to the disease can be selected by univariate analysis (UVA), taking into account the false discovery rate (FDR) [29], and by variables which are important in the projection (VIP) when supervised methods are used [30]. Potential disease biomarkers are further screened as a function of their diagnostic power to discriminate OC patients from healthy individuals by the Receiver–Operating Characteristic (ROC). Besides its diagnostic power, ROC also provides an optimal cut-off value that discriminates the disease condition from the healthy one [31]. The area under the curve (AUC) of the ROC, i.e., ranging from 0.8 to 0.9, provides excellent diagnostic power using single or multiple metabolite biomarkers [21,30,31]. A good diagnostic biomarker has to reach at least 10% of the positive predictive value (PPV), which corresponds to a sensitivity higher than 75% and a specificity higher than 99.6% [32].

The significance of the selected metabolomic variables as diagnostic biomarkers needs to be confirmed by external validation with an independent set of samples [31], and by the investigation of the biochemical mechanisms associated with the identified metabolic biomarker [33]. 

## 3. Metabolomics Diagnostic Biomarkers of OC

The present review only includes studies carried out on humans where case-control groups were suitable for revealing diagnostic biomarkers. Studies that did not consider healthy or benign conditions as controls group were excluded. The quality of the selected studies was assessed by a QUADOMICS tool that is capable of evaluating the design and methodology of the included investigations [17] (Table S1). The percentage of the criteria that scored positively can be considered as an index of quality; however, there is no threshold value that can determine the eligibility of all studies. Nonetheless, all studies included in the present review scored higher than 50% (Table S1).

Table 1 summarizes the selected case-control metabolomics studies. For each study, the biological matrices are reported, together with the applied analytical technique, the adopted statistical methods for biomarkers selection, and the results consisting of metabolomic OC signatures and their diagnostic power and validation. 

### 3.1. OC Specific Metabolomics Signatures for OC

Metabolomic studies have identified a variety of metabolomics signatures which are specific to OC, and therefore, can be used for diagnostic purposes. These signatures involve numerous metabolites that can be grouped into three principal classes, i.e., lipids, amino acids and metabolites belonging to the central metabolism. These metabolic classes are discussed widely due to their potential biological roles in OC development. 

#### 3.1.1. Lipids

Lipids are a heterogeneous class of metabolites involved in many key cellular functions, such as membrane structure building, energy storage and signal transduction. Lipid metabolism is considered to be associated with cell proliferation, inflammation, immunity and apoptosis, and it has been found to be significantly altered in different human cancers [66].

Various specific lipids have been found to be altered in OC patients compared with healthy individuals (Figure S1a–c). In the considered metabolomics studies, OC patients showed an increase of total ceramides (Cer) [38,41,46,52], carnitines [37,44,45] and fatty acids (FA) [42,43,53], and a decrease of triglycerides (TG) [34] and lysophosphatidylcholines (LPC) [28,39,45,49,52,54]. 

The upregulation of blood Cer has been frequently reported in OC studies [38,41,46,52]. A wide MS targeted study, encompassing 232 lipids, revealed a significant increase of certain Cer in EOC patients, i.e., C16, C22, and C24 derivatives [46]. The high levels of these Cer may be associated with the enhanced activity of the ceramide synthases (CerS) CerS6 and CerS2 isoforms that seem to promote tumor growth by acting as apoptosis inhibitors [46,67]. The Cer increase was found to be associated with the corresponding increase of sphingomyelins (SM) [68], in particular, of SM C18 and SM C18:1 [46]. This metabolic feature is interesting, since SM are synthetized from Cer by the sphingosine-1-phosphate pathway (S1P) that is highly expressed in cancer cells; therefore, it is currently under investigation as a potential pharmacological target for OC [69]. The OC diagnostic value of serum C16 and C22 Cer derivatives was remarkable, with an AUC = 0.89, that further improved to 0.92 when they were combined with SM and CA125 [46]. In spite of this diagnostic potential, the study showed some limitations, i.e., the lack of an independent set of samples for metabolite validation and for the analysis of a very small group of patients and controls.

A different untargeted MS study revealed that also hexosyl Cer derivatives, such as galactosylceramide and lactosylceramide, were upregulated in the serum of OC patients [52]. These metabolites were able to distinguish OC from the healthy status with an AUC > 0.75, as confirmed by external validation [52]. Lactosylceramide synthesis is activated in the presence of pro-inflammatory factors in the tumor environment, and leads to the activation of different tumor growth related-signaling pathways involved in proliferation, adhesion, migration and angiogenesis [70]. Moreover, the enzyme responsible for the synthesis of galactosylceramide has been reported as an index of tumor aggressiveness in different cancer types such as breast cancer [71]. This metabolomic evidence suggests an active role of these Cer derivatives in OC development and proliferation.

Carnitines and aceyl-carnitines levels were also found to be high in OC patients in three independent studies [37,44,58]. Only trans-2-dodecenoylcarnitine levels were shown to be low in blood, but its biological significance was not confirmed in an in vivo model [39]. Acyl-carnitines play a key role as carriers of FA into the mitochondria; their accumulation is indicative of oxidative-phosphorylation impairment. Since the acylation state of carnitine in plasma reflects the composition of the cytosolic acylcarnitine pool, it can be used as an indicator of mitochondrial health [72]. In cancer, acyl-carnitines have been found to modulate the switch between glucose and FA metabolism [73]. This biochemical effect seems to be important in OC metastatic processes supplied by the omentum, as demonstrated in the adipocyte-OC cell coculture model [74]. Different targeted and untargeted metabolomics studies seems to confirm such a metabolic switch by detecting high serum levels of free FA in OC patients [42,43,53], in particular, of saturated FA stearic, lauric, myristic and palmitic acids [42,43,53], and unsaturated trans-FA palmitelaidic and linolenic acids [43]. FA contribute to fueling the catabolic pathways necessary for cancer growth and proliferation [75], and their synthesis inhibition has been found to suppress tumor growth [76]. Interestingly, epidemiological studies indicated that dietary intake of saturated FA could be associated with a higher risk of OC [77]. In spite of these significant alterations, the diagnostic value of FA has not been deeply investigated. Only for the FA C16:0 has been reported, i.e., an AUC = 0.7 vs. an AUC = 0.91 for the CA 125 in the same series of patients [53]. 

The high demand of FA for cancer growth may also be sustained by increased TG lipolysis. A significant serum reduction of TG derivatives was observed in one study that involved serous OC patients [34], but such a TG alteration has not been confirmed in other metabolomic investigations. A more consistent alteration instead involves the monoacylglycerols (MG) which are found in reduced levels in plasma of EOC patients [39,45]. The MG could be hydrolyzed into free FA to sustain tumor growth by monoacylglycerol lipase (MGL), whose overexpression has been found to be associated with an aggressive OC phenotype [78].

Among phospholipids, LPC and phosphatidylcholines (PC) were found to be deregulated in OC. With the exception of LPC 16:0 [39] and LPC 18:2 [28,42], that are inconsistently reported to be up- and down-regulated, the overall LPC are generally reduced in OC [28,39,45,49,52,54], while PC were found to be increased only in EOC cells [79] but heterogeneously distributed in OC patients [28,41,42,52]. Tumor stage and the presence of metastasis seems to affect LPC levels [44], suggesting a role in the development of OC, especially in the early stage [39]. Their potential use for OC diagnoses has been partially investigated in only one study, that reported a promising AUC > 0.8 for LPC and PC derivatives [49].

Other classes of lipids such as lysophosphatidylethanolamines (LPE) [28,39,41,45,52], phosphatidylethanolamines (PE) [34,42], phosphatidylinositol (PI) [41], lysophosphatidic acid (LPA) [63], phosphatidylserine (PS) [34], as well as steroids and their derivates [23,28,37,39,41,42,44,45,52,63], were also found to be altered in OC. However, because of the low degree of agreement among studies, their role as diagnostic biomarkers for OC still remains to be defined.

In summary, lipid metabolism was shown to be significantly altered in OC, likely as a consequence of the high energy demands required to sustain tumor cell proliferation and growth [80], as well as to support membrane turnover and changes in molecular morphology [35]. Specific circulatory lipid signatures of Cer, carnitines and FA seem to represent promising candidates as diagnostic biomarkers of OC in view of the wide agreement among several independent studies. Their biosynthetic pathway could represent a novel therapeutic strategy for OC.

#### 3.1.2. Amino Acids and Derivatives

Besides their role in protein metabolism, amino acids (AA) play a key role in many cellular biochemical processes such as biosynthesis, energy production, redox balance maintenance, epigenetic regulation and immune response. Such pathways are all deregulated in cancer [81], and the alteration of the circulating AA represents an effective diagnostic tool for different cancers [82,83] including OC [34,37,38,40,43,44,45,49,54,56,57,58,59,60,61,62,63]. The AA signatures of OC were consistently found in independent studies, making them valuable for diagnostic purposes. A summary of the AA alterations in OC, together with their frequency among studies, is reported in Figure S2. 

The upregulation of the AA derivatives 3-(4-hydroxyphenyl) lactate [39,43,44] and kynurenine in blood [44,45] are a common metabolic feature in OC patients. Conversely, methionine [43,56], histidine [44,45,49,56], alanine [34,40,43], piperine [44,45], 2- piperidinone [44,45,54], tryptophan [43,44,45,54,56], 3-indolepropionic acid [44,45], citrulline [49,56] and threonine [43,56] were most frequently found to be downregulated in the blood of OC patients. The low blood levels of methionine, lysine [57,61] threonine [43,56,57] and valine [43,57] were found to be correlated with a corresponding increase at the tumor site. This observation may suggest a high uptake of such AA in tumor tissue, likely due to the overexpression of AA transporters that occurs in many cancers, including OC [84,85]. This could be also explained by the auxotrophic effect of methionine and other essential AA characteristics of OC [86].

In spite of the identification of this metabolic alteration, the diagnostic power of AA signatures has been partially investigated. Only tissue methionine, together with phosphorylcholine, were used to develop a predictive algorithm which is useful to distinguish low grade EOC from controls, with a AUC of 0.94 and a sensitivity and specificity of 1.00 and 0.94, respectively [59]. However, it is noteworthy that tissue biomarkers present a low applicability compared with blood biomarkers in screening programs.

Among downregulated AA, tryptophan is particularly interesting. Significantly low levels were observed in the blood of EOC patients in many studies [43,44,45,54,56], making it the most consistently reported metabolic signature of OC; however, its diagnostic ability was not investigated independently from other metabolites. When it was evaluated in association with lysoPC(18:3) and lysoPC(14:0) lipids, it contributed to distinguishing EOC from benign ovarian tumors in premenopausal patients with an evident benefit for the early diagnosis of OC [54]. However, the study failed to consider healthy subjects, and the tryptophan/lipids diagnostic model requires further validation to be suitable for clinical screening. The downregulation of circulating tryptophan may be associated with increased catabolism at the tumor level by indoleamine 2, 3-dioxygenases (IDO1 and IDO2) that transforms tryptophan in kynurenine [49]. The latter metabolite was indeed found to be upregulated in the blood of EOC patients [44,45]. Such an imbalance in kynurenine-tryptophan levels leads to immune system suppression by reducing T-cell function [87,88], supporting the application of IDO1 inhibitors in OC treatment [89].

Other AA signatures involving histidine and citrulline were found to be able to distinguish OC patients from healthy individuals with an AUC>0.75 [49,56]. The low citrulline levels frequently observed in OC patients may be attributed to intestinal symptoms at the time of diagnosis [90], since this AA is synthetized in the small intestine by enterocytes, and its blood deprivation reflects a reduced enterocyte mass due to intestinal inflammation [91].

Taken together, these metabolomic results seem to indicate that AA metabolism may be profoundly altered as a consequence of OC. Agreement among studies makes such alterations useful for OC diagnoses, especially in the early stage. However, further investigations are needed, since only a few metabolomic investigations deeply explored and validated their diagnostic potential. 

#### 3.1.3. Central Carbon Metabolites

Central carbon metabolism involves biochemical pathways encompassing the glycolysis, tricarboxylic acid (TCA) cycle and pentose phosphate pathway, which serve to convert glucose into metabolic precursors. Such a series of metabolic reactions is profoundly deregulated in cancers; this is linked to the well-known Warburg effect, that consists of the shift from the TCA cycle to aerobic glycolysis for energy production [81,92,93]. In the context of OC, few metabolomics studies have identified specific signatures associated with this cellular metabolic alteration. Only increases in the levels of maltose in blood [36,43] and lactate in tissue [57,58] have been reported, but these have not been widely confirmed among different studies (Figure S3). Overall, despite central carbon metabolites having a central role in cancer metabolism, highly heterogeneous results are presented, hindering their effective clinical application in OC diagnoses using such metabolites.

#### 3.1.4. Other Metabolites

The metabolic OC biomarkers not included in the previously discussed classes, whose alterations found agreement among studies, are summarized in Figure S4. 

First, 3-hydroxybutyric acid was found to be upregulated in the blood of EOC patients [40,43,48]; this is likely associated with the significant FA oxidation observed in tumor [43]. It is converted into its oxidative metabolite, acetoacetate, by 3-hydroxybutyrate dehydrogenase, which has also been reported to be upregulated in the blood of EOC patients [40,43]. Besides 3-hydroxybutyric acid, other altered metabolites include hypoxanthine [28,45], pseudouridine [44,63,64], hippurate [44,62,64], 2,7,8-Trimethyl-2-(beta-carboxyethyl), and gamma-carboxyethyl hydroxychroman (γ-CEHC) [44,45]. However, only 3-hydroxybutyric acid has been considered as potential diagnostic biomarker. Its diagnostic power, evaluated by ROC analysis, was comparable with that of CA125 within the same series of case-control groups [43], underlying its potential role in predicting OC malignancy.

## 4. Conclusions

The complex interplay between OC and the host brings about metabolic alterations that can be revealed by metabolomics profile analyses. During the last decade, many metabolomic signatures have been proposed as potential biomarkers for the detection of OC in an early stage of development, to improve current diagnostic tests, and to give insights into the development of new targeted interventions. The most promising circulating signatures of OC involve metabolites belonging to lipids and AA pathways. These metabolic fingerprints find agreement in many studies, making them relevant for OC diagnosis. However, their clinical application appears to be limited because a lack of independent, large validation studies prevents their effective use for OC screening and monitoring. Future research should include better designed studies on large homogeneous populations that include proper external validation in order to further improve the translational success of metabolomics for OC diagnosis.

## Figures and Tables

**Table 1:** Metabolomics studies for the discovery of OC diagnostic biomarkers.

Ref	Sample	Analytical Techniques	Test/Control Groups	Data Pretreatment/Statistical Analysis	Specific OC Metabolites	Diagnostic Power *
[34]	Plasma	LC-QTOF	SOC (*n* = 25) BC (*n* = 25) IVS: SOC (*n* = 25) BC (*n* = 25) (d)	Bridge Normalization, Log Transformation, Linear Regression Analysis	**↓C52H79NO5S ↓ PS(O-18:0/0:0) 18:3 ↓ Cholesteryl ester ↓TG(16:0/16:1(9Z)/16:1(9Z))[iso3]** ↓alanine↓PG(P-20:0/12:0) ↓ TG(16:1(9Z)/16:1(9Z)/16:1(9Z))↓PS(O-18:0/16:1(9Z)) ↓TG(17:2(9Z,12Z)/17:2(9Z,12Z)/20:5(5Z,8Z,11Z,14Z,17Z))[iso3] ↓TG(16:1(9Z)/17:2(9Z,12Z)/17:2(9Z,12Z))[iso3]↓TG(16:1(9Z)/17:1(9Z)/17:2(9Z,12Z))[iso6]↓PE(18:1(9Z)/20:3(8Z,11Z,14Z))↓C57H102N2OS ↓TG(16:0/16:0/16:1(9Z))[iso3] ↓ C29H47N9 O2 ↓ PS(O-20:0/0:0) ↓PE(18:1(9Z)/20:3(8Z,11Z,14Z))↓TG(16:1(9Z)/17:0/17:2(9Z,12Z))	TPR = 95%, TNR = 35%, AUC = 0.91 (+CA125)
[35]	Serum, tissue (a)	LC-QTOF/ QQQ	EOC (*n* = 29) BOT (n=28) HC (*n* = 27) IVS: HC (*n* = 361) BOT(*n* = 199), EOC(*n* = 125)	TIC Normalization, Pareto Scaling, Wilcoxon Rank Sum Test, PLS-DA	↑ CPG	TPR = 67%, TNR = 77%, AUC = 0.75
[28]	Serum	LC-QTOF	EOC (*n* = 21) EOC recurrent (*n* = 36) EOC non recurrent (*n* = 25) HC (*n* = 24)	TIC Normalization, Wilcoxon Test, PCA, PLS-DA	↑ Hypoxanthine ↑ Guanidinosuccinic acid ↑ Cortisol ↑ Lyso PE(22:6) ↓ Lyso PC(18:2)	ACC = 88.7%
[36]	Serum (b)	LC–QQQ	OC (*n* = 21) BC (*n* = 17), HC (*n* = 20)	*t*-Test	↑arabitol, **↑maltose, ↑ maltotriose, ↑raffinose, ↑mannitol**, ↓inosine, ↓ ribose, ↑glucose, ↑ (only vs BOT) erythritol	AUC = 0.91 (OC vs HC) AUC = 0.83 (OC vs BC)
[37]	Blood, tissue	GC/LC-MS	OC (*n* = 21) BC (*n* = 16)	ANOVA, Welch’s two-sample *t*-test, Matched Pairs *t* tests.	↑N-acetyl putrescin ↑ N-acetyl tryptophan ↑acyl carnitines	/
[38]	Plasma (a)	LC-QTOF	EOC (*n* = 42) HC (*n* = 58) IVS: EOC (*n* = 38) HC (*n* = 35) (f,c)	Logistic Regression, Wilcoxon Ranksum Test, PLS-DA	↓ Demethylphylloquinone ↑ Ganglioside ↑ Lysophospholipids ↑ Ceramides ↑ Phytosphingosine ↑ N-Formylkynurenine	TPR = 92.1%, TNR = 88.6%, AUC = 0.94
[39]	Plasma (a)	LC-QTOF	EOC (*n* = 21) HC (*n* = 31) (f)	TIC Normalization, Two-Sided Cochran And Cox Test, PCA, OPLS-DA	↓2-Piperidinone↓ 4-(2-Aminophenyl)-2, 4-dioxobutanoic acid↓trans-2-Dodecenoylcarnitine↑ Pipericine↓ MG (18:2)↓ Coniferin ↑ Adrenoyl Ethanolamide (h)↓ LysoPE(16:0)(h)↓ LysoPC(14:0) (h)↑ LysoPC(16:0)↓ Taurochenodesoxycholic acid↓ LysoPC(17:0)↓ LysoPC(18:3) ↓ LysoPE(22:6)	AUC = 0.94
[40]	Serum (a)	NMR	EOC (*n* = 120) HC (*n* = 132) IVS: EOC (*n* = 50) HC (*n* = 50)	Normalization to unit integral, Pareto Scaling, PCA, Logistic Regression	↓alanine↓CH3CH2CH2CO of lipid (mainly in VLDL) ↓CH3(CH2)n of lipid (mainly in LDL), ↓valine ↓ creatine/creatinine ↓choline of phospholipids, ↓CH2CH2CH2CO of lipid (mainly in VLDL) ↓ = CHCH2CH2 of unsaturated lipid ↑β-hydroxybutyrate ↑acetone↑acetoacetate	TPR = 63%, TNR = 80%, AUC = 0.79
[41]	Serum (a)	LC-MS	PSC (*n* = 46) HC (*n* = 43)	Autoscaling, Mann Whitney U Test, OPLS-DA	↑cortisone ↑lysophatidylinositol(18:1) ↑aspartyl-glutamic acid ↓16-(6-butoxy-3-hydroxy-4,5-dimethylcyclohex-1-en-1-yl)-6,10-dihydroxy2,6,10,14-tetramethyl hexadecanoic acid ↑ceramide(d18:1/16:0) ↑lysophosphatidylethanolamine(22:6) ↑2-hydroxyl nonanoic acid ↓iso-1,2-octadecanediol ↓3-hydroxyldodecanedioic acid ↓phosphatidylinositol(20:4/18:1)↓7,9,13-trihydroxyoctacosa-16,22-dienoic acid	TPR = 100%, TNR = 100%
[42]	Serum/ (a)	LC-QTOF	PSC (*n* = 37) BC (*n* = 35)	Linear Intensity Normalization Of The Total Raw Signal	↑12-Hydroxy-8E10E-heptadecadienoic acid ↑palmitic acid ↑stearic acid ↑ GlnHisAla; DHEA sulfate ↑PC(P-16:0/0:0) ↑PC(10:0/4:0) ↑PE(9:0/10:0) ↑ LPC(18:2) ↑PC(14:0/ 20:1(11Z)) ↑PE- NMe(18:1(19E)/18:1(9E)) ↑PC(14:0/22:4 (7Z,10Z,13Z,16Z)) ↑ PC(14:0/22:1(13Z))	/
[43]	Serum, tissue (a,b)	GC×GC-TOF	HGSOC (*n* = 158) BC (*n* = 100)	IS Normalization, Log Transformation, Autoscaling Unpaired *t*-Test, ANOVA, Correlation Analysis (Pearson And Spearman), PCA	**↑3,4-dihydroxybutyric acid ↑3-hydroxybutyric acid**↑ 2-hydroxybutyric acid↑ ↑acetoacetic acid ↑ 2,4-Dihydroxybutyric acid ↑ glycine ↑glutamic acid ↑ glutamine ↑ 4-hydroxyphenyllactic acid ↑ maltose ↑turanose ↑melibiose ↑ mannonic acid ↑ xylitol ↑ arabinose ↑ glucopyranose ↑ mannopyranose ↑xylose ↑ linolenic acid (C18:3) ↑ Palmitelaidic acid (C16:1) ↑ oleic Acid (C18:1) ↑ myristic acid (C14:0) ↑ myristoleic acid (C14:1) ↑ palmitic acid (C16:0) ↑ lauric acid (C12:0) ↑ adipic acid ↑Myo- Inositol ↑ethanolamine ↑glycerol ↑ 3-hydroxyvaleric acid ↑erythritol ↑ maleic acid ↑ 3-hydroxyisovaleric acid ↑ 3-hydroxycaproic acid↓ Tryptophan alanine↓ methionine↓ threonine ↓ proline ↓ serine ↓ phenylalanine; tyrosine ↓valine ↓ 2-oxo-3-methylpentanoic acid ↓indole-3-acetic acid ↓ 2-oxoisovaleric acid ↓2-hydroxy-3-methylvaleric acid ↓ lactic acid ↓ malic acid ↓ glycerol-3-phosphate ↓ 2,3,4-trihydroxybutyric acid ↓ ketoleucine ↓ cholesterol ↓ 2-aminobutyric acid ↓ glyceric acid	AUC = 0.98, AUC = 0.912 (+CA125)
[44]	Plasma	LC-QTOF	EOC (*n* = 80) BOT (*n* = 80) UF(*n* = 80) IVS: EOC (*n* = 60), BOT (*n* = 78) UF (*n* = 70) (f)	Kruskal–Wallis Rank Sum Test, PCA, PLS-DA	↑ 3,5-Tetradecadiencarnitine ↑ cis-5-Tetradecenoylcarnitine ↑Dodecanoylcarnitine ↑ Elaidic carnitine ↑ Tetradecanoylcarnitine ↑ trans-2-Dodecenoylcarnitine ↓ L-Tryptophan ↓ 3-Indolepropionic acid ↑ Kynurenine ↓ 5-Hydroxyindoleacetaldehyde ↓ 3b-Hydroxy-5-cholenoic acid ↓ Glycoursodeoxycholic acid ↓ Deoxycholic acid ↓Androsteroneglucuronide ↓ Prasterone sulphate ↓ 2-piperidinone ↑ PhePhe ↑ L-(-)-3-Phenyllactic ↓ Piperine↓ Hippuric acid ↑ L -Tyrosine ↑ Hydroxyphenyllactic acid ↓ L-histidine ↓ g-CEHC ↓ d-CEHC ↑ Pseudouridine	AUC = 0.91 (EOC vs. BOT) AUC = 0.94 (EOC vs. UF)
[45]	Plasma (a)	LC-QTOF	EOC (*n* = 35) HC (*n* = 55) (c,f)	Welch’s *t*-Test, PCA,PLS-DA	↓LysoPC(8:2) ↓LysoPC(18:3) ↓ LysoPC(14:0) ↓LysoPC(18:1) ↓LysoPC(16:1) ↓ LysoPC(22:5) ↓LysoPC(20:3) ↓ LysoPC(20:4) ↓ LysoPE(18:2/0:0) ↓ LysoPE(0:0/18:1) ↓ L-Tryptophan ↑Kynurenine↓ 3-Indolepropionic acid ↓ Tetracosahexaenoic acid ↓Aldosterone ↓Glycoursodeoxycholic acid ↑Hypoxanthine ↓MG(24:6/0:0/0:0) ↓2-Octenoylcarnitine ↓Piperine ↓2-Piperidinone ↓Retinolc ↓Dodecanedioic acid d ↑L-Thyroxine ↓12,13-DiHODE ↓ 2-Octenoic acid ↓19,20-DiHDPA ↓N-Undecanoylglycine ↓γ-CEHC ↓p-Salicylic acid ↓Nutriacholic acid ↓Deoxycholic acid ↑Hydroxyphenyllactic acid ↑4-Nitrophenol ↓L-Histidine ↓2,6-Dihydroxybenzoic acid ↓β-Hydroxyisovaleric acid	/
[46]	Serum (b)	LC-QQQ	EOC (*n* = 15) BC (*n* = 21), HC(*n* = 21) (c)	Batch Normalization, Log Transformation, Pareto Scaling, PLS-DA	↑Cer 34:1;2 (C16),↑Cer 40:1;2 (C22),↑ Cer 42:1;2 (C24),↑ SM 36:0;2 and SM 36:1;2 (C18 and C18:1)	TPR = 83%, TNR = 87%, AUC = 0.92
[47]	Plasma/breath	Canines, GC-MS ssDNA-CNT sensors	EOC (*n* = 10) BC (*n* = 10) HC (*n* = 10) (c)	/	↓ 3,4-dimethylbenzaldehyde	TPR = 82.5%
[48]	Serum (a)	NMR	EOC (*n* = 38) BC (*n* = 12) HC (*n* = 51)	TIC Normalization, Pareto Scaling, PCA, SIMCA	EOC vs HC (pre and post menopausal) **2.77ppm**,**2.04ppm regions** ↑3-hydroxy-butyrate, EOC vs HC (premenopausal)↑2.25 (unassigned), EOC vs HC (postmenopausal)↑3.7 ppm (sugar hydrogens)	AUC = 1.0
[49]	Serum/ (b)	LC-QQQ	EOC (*n* = 26) BOT (*n* = 25) HC (*n* = 25) (c)	Autoscaling, One Way ANOVA, Kruskal–Wallis Test	↓histidine ↓citrulline ↓ **lysoPC a C16:1, ↓PC aa C32:2, ↓PC aa C34:4 ↓PC aa C36:6**	AUC >0.8
[50]	Serum (a)	LC- Orbitrap	EOC (*n* = 51) HC (*n* = 35) IVS: EOC (*n* = 34) HC (*n* = 25)	*t*-Test, OPLS-DA	↑ 471.73720 m/z	TPR = 98%, TNR = 97%
[51]	Plasma (a)(b)	LC -QQQ	EOC (*n* = 117) HC (*n* = 27) (f)	*t*-Tests, Wilcoxon Rank Sum Test, ANOVA, Logistic Regression Analysis	↑**16:0-LPA** ↑18:0-LPA↑ 18:1-LPA ↑ 18:2-LPA ↑**20:4-LPA** ↑22:6-LPA ↑ 18:0-A-LPA ↑16:0-An-LPA ↑18:0-An-LPA ↑18:0-An-LPA ↑Total A-LPA ↑Total LPA ↑16:0-LPA/20:4-LPA ↑16:0-LPI ↑18:0-LPI ↑20:4-LPI↑↑ S1P	TPR = 91.1%, TNR = 96.3%
[52]	Serum (a)	LC-QTOF	EOC (*n* = 30) HC (*n* = 30) IVS: EOC (*n* = 17) HC (*n* = 18) (c,f)	*t*-Test, PCA, PLS-DA	↓**2-piperidinone**, ↑ eicosadienoic acid, ↑ 7-ketodeoxycholic acid, ↑varanic acid, ↑ **1-heptadecanoylglycerophosphoethanolamine**, ↓2-hexaprenyl-3-methyl-6-methoxy-1, 4benzoquinone, ↓ lysoPC (15:0), ↓lysoPE (0:0/20:0), ↑DG (14:0/20:0/0:0), ↑PC (14:1/22:2), ↑galabiosylceramide (d18:1/18:1),↑ lactosylceramide(d18:1/22:0).	TPR=96.7%, TNR = 66.7%, AUC = 0.89
[53]	Serum (b)	GC-MS	EOC (*n* = 40) HC (*n* = 35) IVS: EOC (*n* = 30) HC (*n* = 30) (c,f)	Autoscaling, *t*-Test, PCA, PLS-DA, Logistic Regression	↑EFA C16:0; C18:0 ↓FFA C16:0	TPR = 62.9%, TNR = 62.9%, AUC >0.68
[54]	Plasma (a)	LC-QTOF	EOC (*n* = 50) BOT (*n* = 50) IVS: EOC (*n* = 30) BOT (*n* = 40) (c) (f)	TIC Normalization, Kruskal–Wallis Rank Sum Test, PLS-DA	↓L-Tryptophan ↓LysoPC(18:3), ↓LysoPC(14:0) ↓ 2-Piperidinone	AUC = 0.84
[55]	Serum (a)	DART-TOF	SPC (*n* = 44) BC,HC (*n* = 50)	Relative intensity normalization, ANOVA, PLS-DA	↑ histamine, alanine, serine, cysteine, threonine, glycine	TPR = 100%, TNR = 98%, ACC = 99%
[56]	Serum/ (b)	LC-QQQ	EOC (*n* = 38) BOT (*n* = 62) HC (*n* = 50) (c,f)	Normalization By Sum, Log Transformation, Auto Scaling, *t*-Test, Welch’s *t*-Test, Mann-Whitney U Test, ANOVA, PLS-DA	↑_α-aminoadipic acid ↓asparagine ↓**citrulline** ↑cystine, ↓glutamine ↓**histidine** ↑isoleucine ↓lysine ↓methionine ↓ornithine ↓threonine ↓ tryptophan	TPR>71%, TNR>72%, AUC>0.76
[57]	Cyst fluid (a,b)	NMR	OC (*n* = 12) BC (*n* = 28)	Mann–Whitney U test	↑ isoleucine ↑ valine ↑ threonine ↑lactic acid ↑lysine ↑ methionine ↑ glutamine ↑choline	/
[58]	Tissue	LC-MS/MS, GC-MS	EOC (*n* = 18) HC (*n* = 12) (c,f)	ANOVA, PCA	↓ 1-methylimidazole acetate ↓taurine ↓phenol sulfate ↓ 6-phosphogluconate↑betaine ↑carnitine ↑malate ↑ fumarate ↑ N-acetylglycine ↑lactate ↑fucose ↑acetylcarnitine ↑butyrylcarnitine ↑propionylcarnitine ↑2aminobutyrate ↑N-acetylasparate (NAA) ↑Nascetyl- aspartyl-glutamate (NAAG)	/
[59]	Tissue (b)	NMR, MS	LGSOC (*n* = 15) HGSOC (*n* = 19) BC (*n* = 17)	Autoscaling, Log Transformation *t*-Test,Mann–Whitney U Test, ANOVA, PCA, PLS-DA, Logistic Regression	LGEOC: ↓phosphoryl choline ↑methionine / HGEOC: ↑PC ae C38:2 ↑PC aeC32:2 (from predictive model)	TPR > 95%, TNR >94%, AUC >0.92
[60]	Cyst fluid, serum (b)	GC-MS	EOC (*n* = 25) BOT (*n* = 36)	Mann U Whitney, Kruskal Wallis Tests, Spearman’s Rank Correlation Testing	↑ NAA	/
[61]	Cyst fluid (a)	NMR	EOC (*n* = 10) BOT (*n* = 8)	Probabilistic Quotient Normalization, Centering, Mann-Whitney Tests, PCA	↓citrate↑ lysine	/
[62]	Urine	NMR	EOC (*n* = 40) HC (*n* = 62) IVS: EOC (*n* = 10) HC (*n* = 10)	Probabilistic Quotient Normalization, Log transformation, Autoscaling, Wilcoxon’s Rank-Sum Test, PCA, PLS-DA, OPLS-DA	↓ Creatine ↓ Acetate ↓Succinate ↓Levoglucosan ↓ Lactate ↓Pyroglutamate ↓Formate ↓Isoleucine ↓Sucrose ↓ Trigonelline ↓Leucine ↓Asparagine ↓Urea ↓Glucose ↓Ethanolamine ↓Dimethylamine ↓4-Hydroxyphenylacetate ↓Creatinine ↓Alanine ↓Hippurate ↓1-Methylnicotinamide ↓Uracil ↓Valine ↓trans-Aconitate ↓methanol	TNR = 99%
[23]	Urine (a,b)	GC- LC-MS	OC (*n* = 4) HC (*n* = 8) (e)	Normalization to creatinine / ANOVA, PLS-DA	↑ 4-androstene-3,17-dione ↑ 1-methyl-adenosine ↑ 3-methyluridine	/
[63]	Urine (a)	LC-QTOF	EOC (*n* = 58) BOT (*n* = 62) HC (*n* = 54)	TIC normalization, Kruskal−Wallis Rank Sum Test, PCA, PLS-DA	↑imidazol-5-yl-pyruvate ↓L-histidine ↓prasterone sulfate ↑N-acetylneuraminate 9-phosphate ↑N4-acetylcytidine ↑urate-3ribonucleoside ↑pseudouridine ↓**Nα-acetyl-L-glutamine** ↑succinic acid ↑**(S)-reticuline** ↑N-acetylneuraminic acid ↑**3-sialyl-N-acetyllactosamine** ↑β-nicotinamide mononucleotide ↑prolylhydroxyproline ↑**selenocystathionine** ↑ **3-sialyllactose**↑N-acetylgalactosamine 4-sulfate ↓glycodeoxycholate ↑**3-dehydroquinic acid ↑3-indolelactic acid↑LPA(P-16:0e/0:0)**	AUC = 0.728, AUC = 0.87 (+ CA125)
[64]	Urine (a)	LC-QTOF	EOC (*n* = 22) BOT (*n* = 29) HC (*n* = 25)	Intensity signal normalization, *t*-Test, PCA, OPLS-DA	↑pseudouridine, ↑phytosphingosine, ↓hippuric acid, and↓ homovanillic acid sulfate	/
[65]	Breath (b)	GC-MS, nanoarray	EOC (*n* = 28) BC (*n* = 60) HC (*n* = 34) IVS: EOC (*n* = 12) BC (*n* = 26) HC (*n* = 14) (f)	ANOVA, DFA	↑decanal;↑nonanal; ↑styrene↑2-butanone;↑hexadecane	TRP>73%, TNR>77%, ACC >75%

(a) Untargeted analysis; (b) quantitative MS analysis; (c) no previous cancer treatments; (d) postmenopausal; (e) premenopausal; (f) no metabolic, liver, or kidney pelvic diseases. * parameters refer to multivariate ROC curve of metabolites (in bold metabolites diagnostic power parameters refer to them). The metabolites used to build the diagnostic model are indicated by bold font,↑up-regulated metabolite ↓down-regulated metabolite. HGSOC high-grade serous ovarian cancer; LGSOC low-grade serous ovarian cancer; EOC epithelial ovarian cancer; UF uterine fibroids; PSC Papillary serous carcinoma; HC healthy control; BC benign condition; BOT benign ovarian tumors; IVS independent validation set. NAA *N*-acetyl-l-aspartic acid; CPG 27-nor-5beta-cholestrane-3,7,12,24,25 pentol glucuronide; FFA free fatty acid; EFA esterified fatty acid; VOCs Volatile Organic Compounds. TPR true positive ratio (sensitivity), TNR true negative ratio (specificity), ACC accuracy; DFA Discriminant Function Analysis; SIMCA soft independent modelling by class analogy; CV cross validation; LOOCV leave one out cross validation; DART Direct analysis in real time.

## Supplementary Materials

The following are available online at https://www.mdpi.com/2218-1989/10/10/419/s1, Figure S1: Identified altered lipids metabolites, Figure S2: identified altered AA metabolites, Figure S3: identified altered central carbon metabolites, Figure S4: other metabolites identified as significantly altered, Table S1 *QUADOMICS* evaluation of selected studies.
