# Supplementary material for: Emerging Role of Metabolomics in Ovarian Cancer Diagnosis
Source: Metabolites. 2020 Oct 19;10(10):419. doi: 10.3390/metabo10100419 (PMC7603269; doi:10.3390/metabo10100419)
Supplement: Supplementary file 1 [file metabolites-10-00419-s001.pdf]

Table S1. QUADOMICS studies evaluation

List of queries: 1) Were selection criteria clearly described?; 2) Was the spectrum of patients representative of patients who will receive the test in practice?; 3) Was the type of sample fully described?; 4)Were the procedures and timing of biological sample collection with respect to clinical factors described with enough detail? 4a) Clinical and physiological factors, 4b) Diagnostic and treatment procedures; 5) Were handling and pre-analytical procedures reported in sufficient detail and similar for the whole sample? And, if differences in procedures were reported, was their effect on the results assessed?; 6) Is the time period between the reference standard and the index test short enough to reasonably guarantee that the target condition did not change between the two tests?; 7) Is the reference standard likely to correctly classify the target condition?; 8) Did the whole sample or a random selection of the sample receive verification using a reference standard of diagnosis?; 9) Did patients receive the same reference standard regardless of the result of the index test?; 10) Was the execution of the index test described in sufficient detail to permit replication of the test?; 11) Was the execution of the reference standard described in sufficient detail to permit its replication?; 12) Were the index test results interpreted without knowledge of the results of the reference standard? ; 13) Were the reference standard results interpreted without knowledge of the results of the index test?; 14) Were the same clinical data available when test results were interpreted as would be available when the test is used in practice?; 15)Were interpretable / intermediate test results reported?; 16)Is it likely that the presence of over-fitting was avoided?
Assignment: Y= criteria achieved, N= criteria not achieved, N/A= not applicable.

Reference	1	2	3	4a	4b	5	6	7	8	9	10	11	12	13	14	15	16	% yes
[53]	N	N/A	Y	Y	Y	Y	Y	Y	Y	Y	Y	Y	N	Y	N/A	N	Y	86
[65]	Y	N/A	Y	Y	Y	Y	Y	Y	Y	Y	N	Y	N	Y	N/A	N	Y	80
[47]	N	N/A	Y	Y	Y	Y	Y	Y	Y	Y	N	Y	N	Y	N/A	N	N	71
[46]	Y	N/A	Y	Y	Y	Y	Y	Y	Y	Y	Y	Y	N	Y	N/A	N	N	80
[49]	N	N/A	Y	Y	Y	Y	Y	Y	Y	Y	Y	Y	N	Y	N/A	N	Y	86
[52]	N	N/A	Y	Y	Y	Y	Y	Y	Y	Y	Y	Y	N	Y	N/A	N	Y	86
[37]	N	N/A	N	N	N	N	Y	Y	Y	Y	N	Y	N	Y	N/A	N	?	50
[39]	Y	N/A	Y	Y	Y	Y	Y	Y	Y	Y	Y	Y	N	Y	N/A	N	Y	87
[41]	N	N/A	Y	N	N	Y	Y	Y	Y	Y	Y	Y	N	Y	N/A	N	N	75

[51]	N	N/A	Y	Y	Y	Y	Y	Y	Y	Y	N	Y	Y	Y	N/A	N	N	79
[48]	N	N/A	Y	Y	N	Y	Y	Y	Y	Y	Y	Y	N	Y	N/A	N	N	77
[54]	Y	N/A	Y	Y	Y	Y	Y	Y	Y	Y	Y	Y	N	Y	N/A	Y	Y	93
[40]	N	N/A	Y	Y	N	Y	Y	Y	Y	Y	Y	Y	N	Y	N/A	Y	Y	86
[55]	N	N/A	Y	Y	N	Y	Y	Y	Y	Y	N	Y	N	Y	N/A	N	Y	71
[45]	N	N/A	Y	Y	N	Y	Y	Y	Y	Y	Y	Y	N	Y	N/A	N	N	71
[44]	N	N/A	Y	Y	Y	Y	Y	Y	Y	Y	Y	Y	N	Y	N/A	N	Y	86
[38]	N	N/A	Y	Y	Y	Y	Y	Y	Y	Y	Y	Y	N	Y	N/A	N	Y	86
[42]	N	N/A	Y	Y	Y	Y	Y	Y	Y	Y	Y	Y	N	Y	N/A	N	N	79
[50]	N	N/A	Y	Y	Y	Y	Y	Y	Y	Y	N	Y	N	Y	N/A	N	Y	79
[28]	N	N/A	Y	N	N	Y	Y	Y	Y	Y	Y	Y	N	Y	N/A	N	N	64
[35]	Y	N/A	Y	Y	Y	Y	Y	Y	Y	Y	Y	Y	N	Y	N/A	N	Y	87
[62]	N	N/A	Y	Y	N	Y	Y	Y	Y	Y	N	Y	N	Y	N/A	N	Y	71
[64]	N	N/A	y	N	N	Y	Y	Y	Y	Y	Y	Y	N	Y	N/A	N	N	64
[23]	N	N/A	Y	Y	Y	Y	Y	Y	Y	Y	Y	Y	N	Y	N/A	Y	N	86
[63]	N	N/A	Y	Y	N	Y	Y	Y	Y	Y	Y	Y	N	Y	N/A	N	N	71

[58]	N	N/A	Y	N	N	Y	Y	Y	Y	Y	Y	Y	N	Y	N/A	N	N	64
[43]	N	N/A	Y	Y	Y	Y	Y	Y	Y	Y	Y	Y	N	Y	N/A	Y	N	86
[36]	N	N/A	Y	Y	Y	Y	Y	Y	Y	Y	Y	Y	N	Y	N/A	N	N	79
[61]	N	N/A	Y	N	N	Y	Y	Y	Y	Y	Y	Y	N	Y	N/A	Y	N	71
[60]	N	N/A	Y	Y	N	Y	Y	Y	Y	Y	Y	Y	N	Y	N/A	N	N	71
[57]	N	N/A	Y	N	N	Y	Y	Y	Y	Y	N	Y	N	Y	N/A	N	N	57
[34]	N	N/A	Y	Y	Y	Y	Y	Y	Y	Y	Y	Y	N	Y	N/A	N	Y	86
[59]	N	N/A	Y	Y	Y	Y	Y	Y	Y	Y	Y	Y	?	?	N/A	N	N	83
[44]	N	N/A	Y	Y	Y	Y	Y	Y	Y	Y	Y	Y	N	N	N/A	N	N	71

Figure S1. Frequency of the diagnostic metabolites for OC reported in metabolomic studies for (**a,b,c**) lipids.

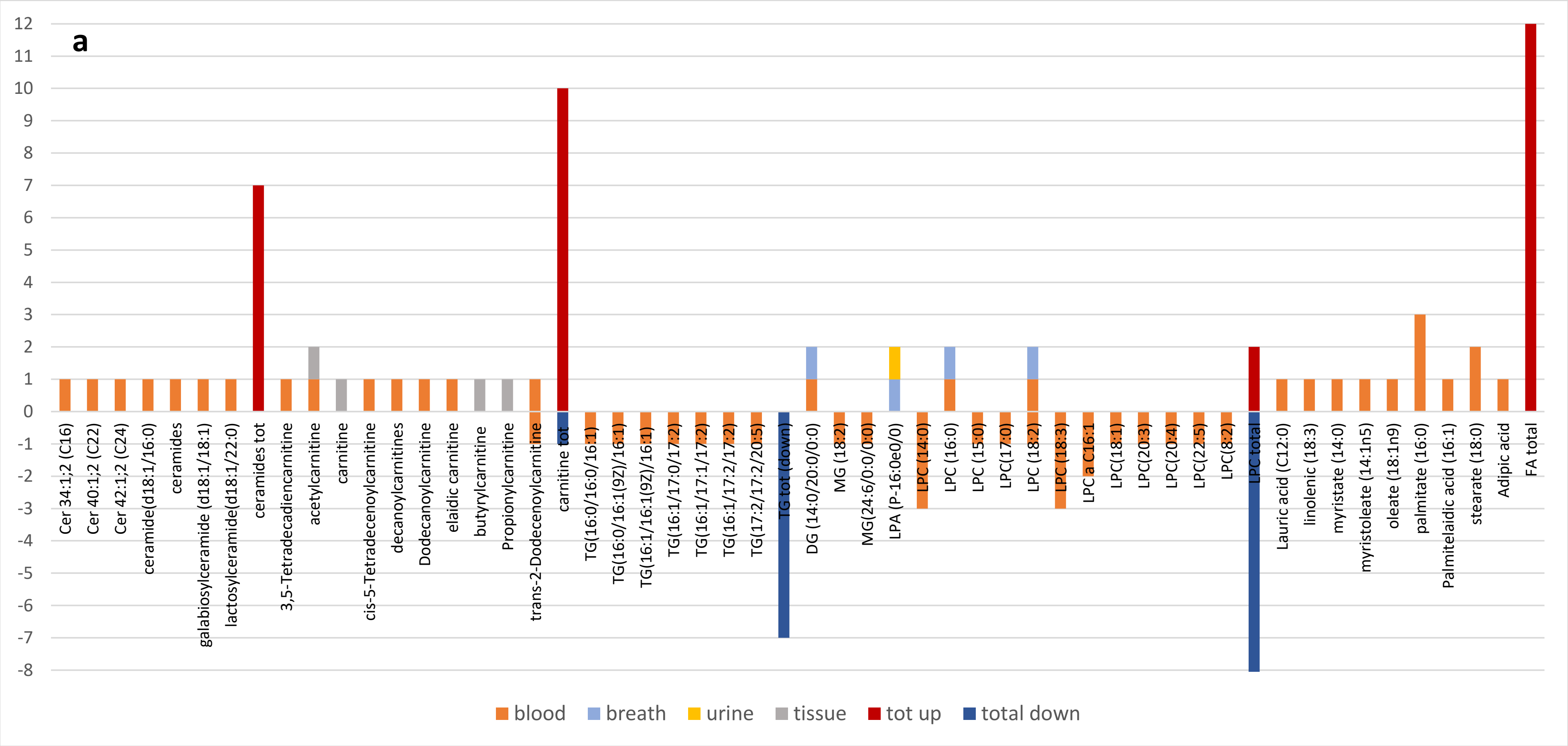

*Positive and negative values of frequency on the y-axes indicate metabolites found as upregulated or downregulated respectively.

Fig.S1 b, c. Lipids class

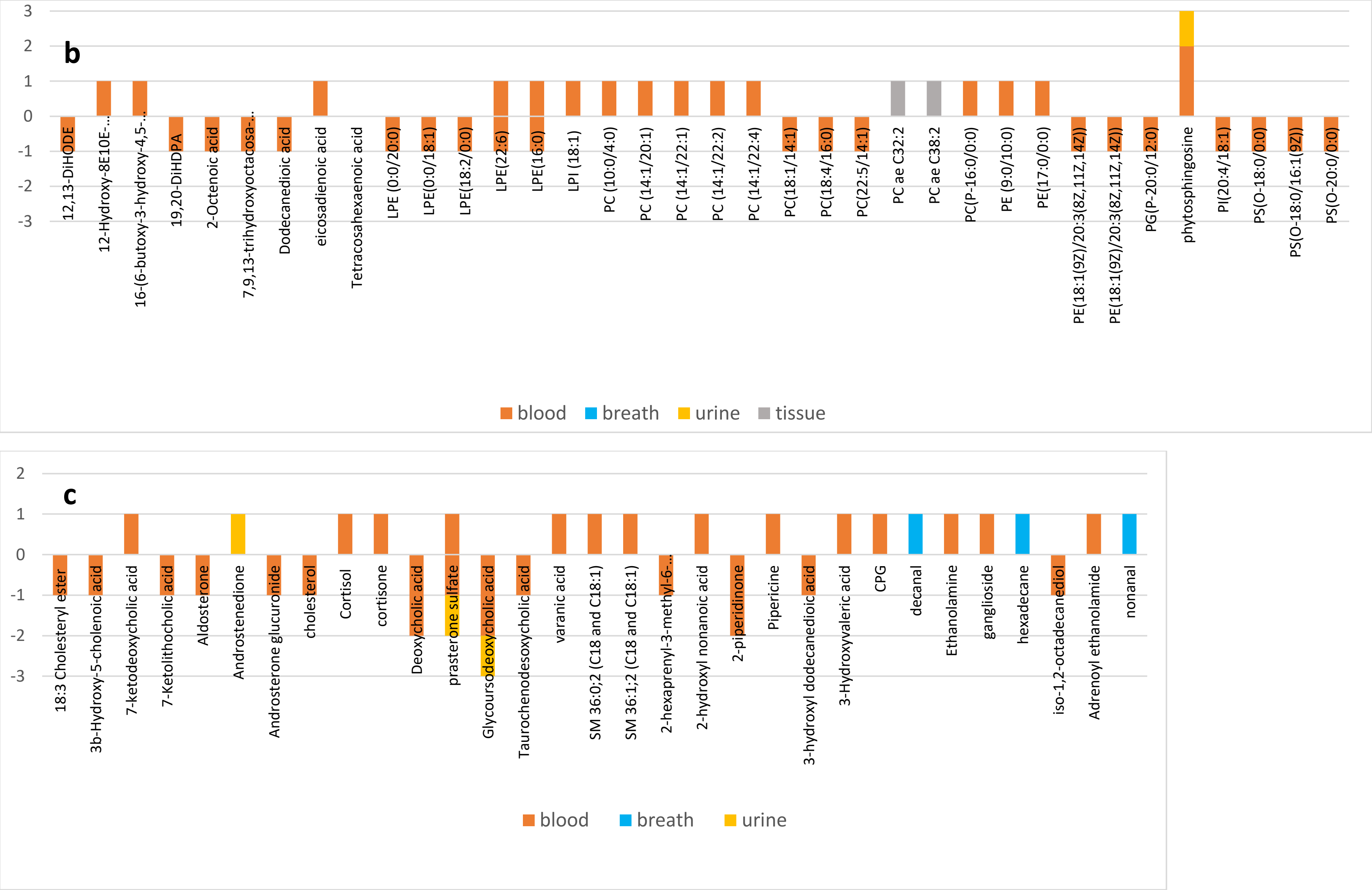

Figure S2. Frequency of the diagnostic metabolites for OC reported in metabolomic studies for amino acids and derivatives.

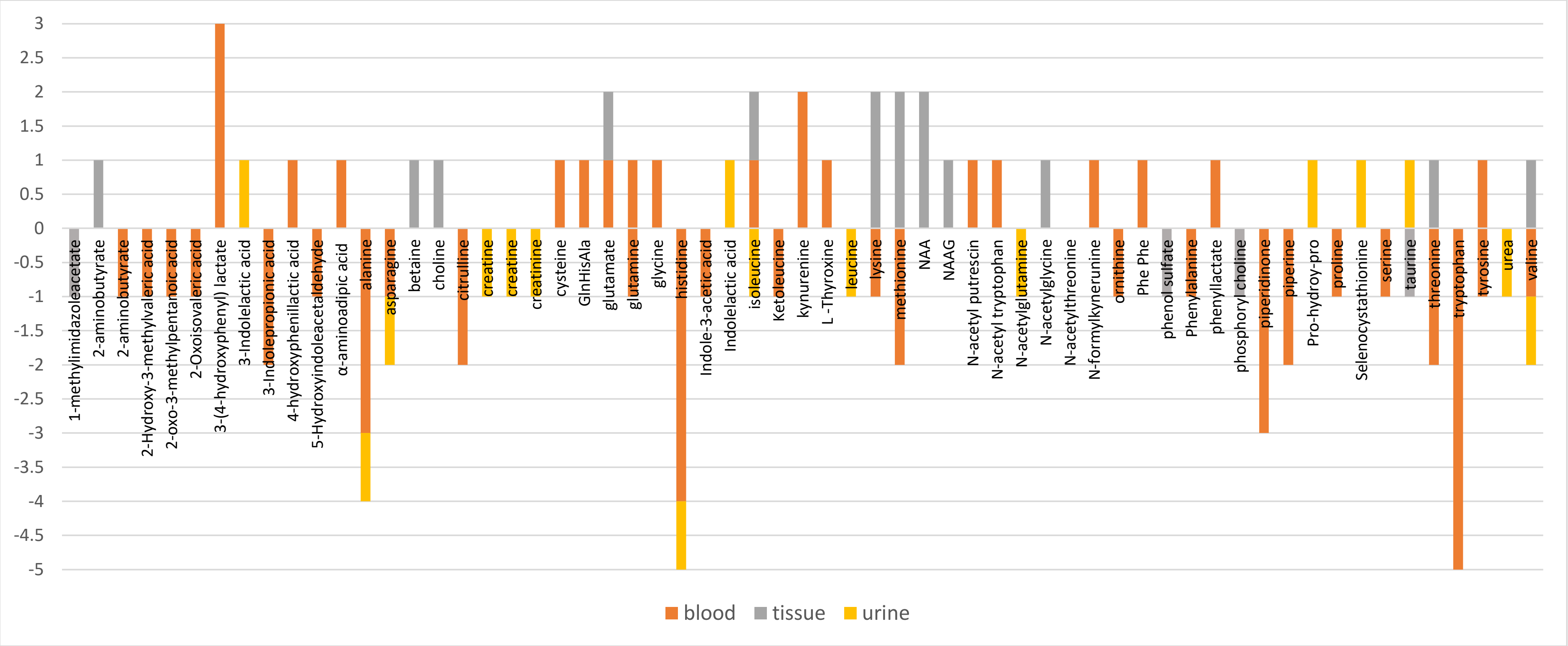

*Positive and negative values of frequency on the y-axes indicate metabolites found as upregulated or downregulated respectively.

Figure S3. Frequency of the diagnostic metabolites for OC reported in metabolomic studies for carbon central metabolites.

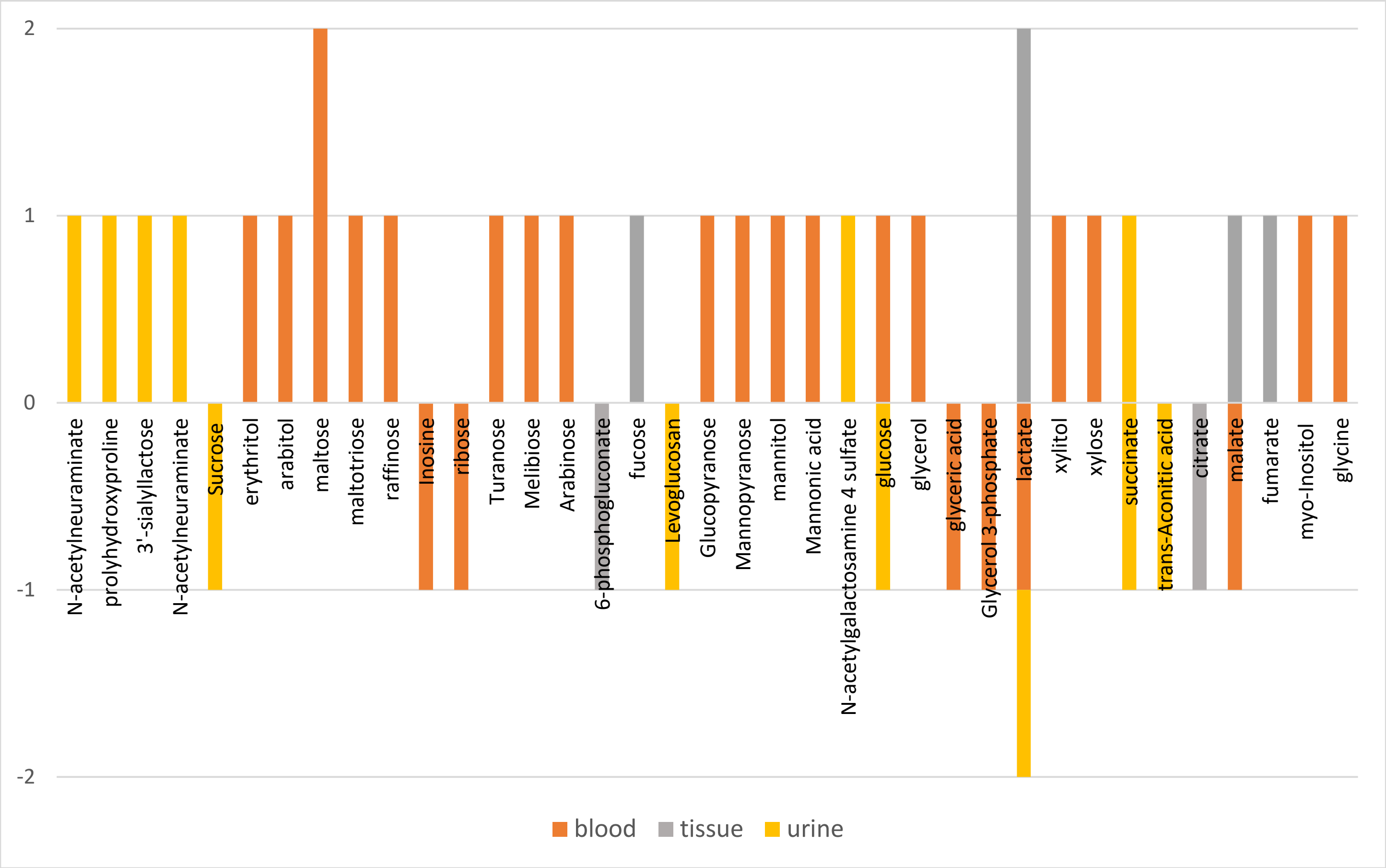

* Positive and negative values of frequency on the y-axes indicate metabolites found as upregulated or downregulated respectively.

Figure S4. Frequency of the diagnostic metabolites for OC reported in metabolomic studies for other metabolites.

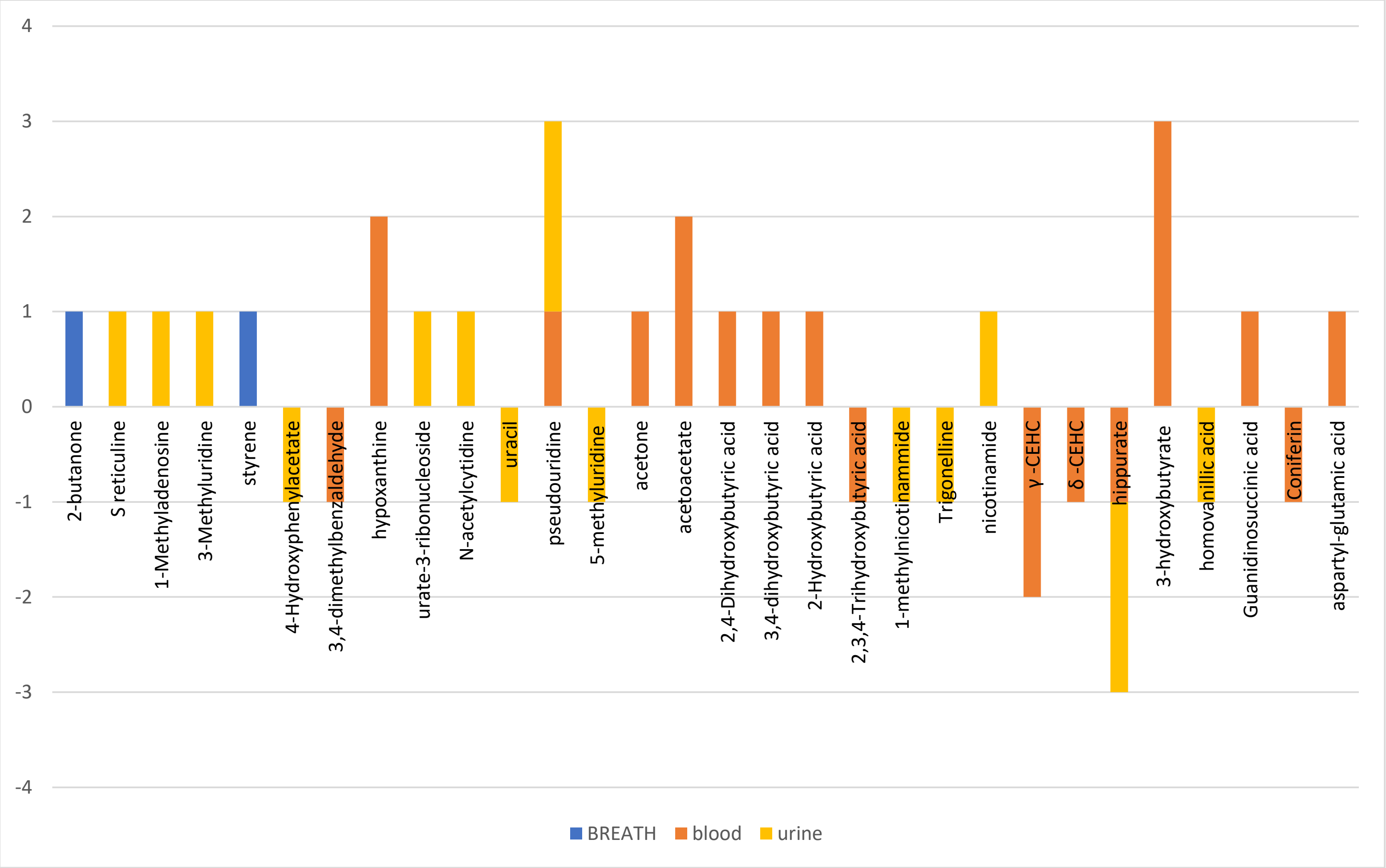

* Positive and negative values of frequency on the y-axes indicate metabolites found as upregulated or downregulated respectively.
